# The Click Production of Captive Yangtze Finless Porpoises (*Neophocaena asiaeorientalis asiaorientalis*) Is Influenced by Social and Environmental Factors

**DOI:** 10.3390/ani11020511

**Published:** 2021-02-16

**Authors:** Agathe Serres, Chen Xu, Yujiang Hao, Ding Wang

**Affiliations:** 1 Institute of Hydrobiology, Chinese Academy of Sciences, Wuhan 430000, China; wangd@ihb.ac.cn; 2 University of Chinese Academy of Sciences, Beijing 100864, China; 3 School of Naval Architecture and Ocean Engineering, Huazhong University of Science and Technology, Wuhan 430000, China; m201871613@hust.edu.cn

**Keywords:** click rate, emotional state, enrichment, noise, social separation, visitors, vocalizations, welfare

## Abstract

**Simple Summary:**

Yangtze finless porpoises’ high-frequency clicks have often been studied and used for wild population surveys. However, the influence of captive environmental and social variables on Yangtze finless porpoises’ production of such signals has never been investigated. In the present study, the click production of a group of captive Yangtze finless porpoises was analyzed across various contexts. This click production was significantly impacted by temporal factors (season), social factors (social separation), and environmental factors (training sessions, presence of enrichment, noise, presence of visitors). The patterns found in this study may be useful for further monitoring of the welfare of captive groups of Yangtze finless porpoises (e.g., welfare assessments) as well as for improving wild surveys (e.g., more accurate interpretation of click density).

**Abstract:**

Yangtze finless porpoises use high-frequency clicks to navigate, forage, and communicate. The way in which click production may vary depending on social or environmental context has never been investigated. A group of five captive Yangtze finless porpoises was monitored for one year, and 107 h of audio recordings was collected under different conditions. Using a MATLAB-generated interface, we extracted click density (i.e., number of clicks per minute) from these recordings and analyzed its variation depending on the context. As expected, click density increased as the number of animals present increased. The click density did not exhibit diurnal variations but did have seasonal variations, with click density being highest in summer and fall. Yangtze finless porpoises produced more clicks when socially separated than when not (136% more), during training/feeding sessions than outside of such sessions (312% more), when enrichment was provided (265% more on average), and when noisy events occurred rather than when no unusual event occurred (22% more). The click density decreased when many visitors were present in the facility (up to 35% less). These results show that Yangtze finless porpoises modulate their click production depending on the context and suggest that their echolocation activity and their emotional state may be linked to these changes. Such context-dependent variations also indicate the potential usefulness of monitoring acoustical activity as part of a welfare assessment tool in this species. Additionally, the click density variation found in captivity could be useful for understanding click rate variations of wild populations that are hardly visible.

## 1. Introduction

Odontocetes produce acoustic signals, including whistles, clicks, and burst-pulsed calls [1]. Whistles are frequency-modulated, long-duration tonal calls, which often have harmonic components [2]. Clicks are directional signals, normally of high frequency [3], and burst-pulsed signals are click trains emitted with very short inter-click intervals [4]. Odontocetes are known to produce clicks more than they produce other types of sounds, probably due to the higher energetic costs of whistles in comparison to clicks [5]. Echolocation is a sensory modality used by odontocetes to obtain an assessment of their environment, consisting of emitting clicks and receiving the echoes from the clicks reflecting off objects. Odontocetes use echolocation clicks to explore their environment (including locating objects and prey) and as navigation cues [3,6]. Even though these clicks are assumed to be mainly produced for echolocation purposes, they have also been suggested to be used for social communication by some species [7,8,9].

Researchers may be able to obtain information about marine mammals’ behavior or their environment based on the specific types of sounds they emit and the variations in call rates [10,11,12]. Some odontocete species, including porpoises (*Phocoenidae* family [13,14,15,16,17,18,19]), Commerson’s dolphins (*Cephalorhynchus commersonii* [20,21,22,23,24,25]), Hourglass dolphins (*Lagenorhynchus cruciger* [21,22]), and pygmy sperm whales (*Kogia breviceps*, [8]), are thought to only produce narrowband and high-frequency clicks, but not whistles. Click emission rate variations would therefore represent the only way for these animals to communicate [7,9,26]. The variability of recorded click rates can reflect periods of activity and rest [27,28,29,30]. Understanding the context of click production might be useful when studying wild animals’ movement patterns; for example, a lack of acoustic detections may not reflect the absence of animals, but rather, result from the presence of non-vocal animals [31]. Lima et al. [32] suggested that more studies on the context of acoustic signal production were needed to understand free-ranging odontocete behavior when animals are not visible. Assessments of odontocetes’ responses to stimuli that are potential disturbances usually focus on whether animals leave exposed areas [33,34]. However, animals that do not leave can exhibit changes in activity budgets [35,36]. Monitoring such changes has often been suggested to be partly possible through the analysis of these animals’ echolocation activity [37,38]. Knowledge of condition-dependent acoustic production rates in small odontocetes is still limited [39]. Authors have suggested that more contexts should be investigated to study the presence of referential content in the acoustic production of odontocetes [40,41,42,43].

Vocal activity has been suggested to be a valid non-invasive tool for monitoring the influence of captive environments and daily routines on farm and zoo animals (e.g., [44,45,46,47,48,49,50,51]). However, the potential use of this method as a tool to assess captive odontocetes’ welfare has rarely been studied [42,43,52]. Monitoring the acoustic activity of these animals might provide valuable information about their daily rhythm, health, and welfare [42,43,52,53]. Understanding captive animals’ vocal activity patterns could aid in improving their management under human care [32]. Using acoustic activity to monitor odontocetes is convenient and less time-consuming than behavioral coding. Analyzing click rates can be automated and therefore achieved relatively quickly. In addition, placing sound-recording devices in the water is non-invasive for animals, who can habituate to these objects. Such passive acoustical monitoring would allow caretakers to notice changes in baseline patterns, supporting a closer examination of the animals.

Yangtze finless porpoises (*Neophocaena asiaeorientalis asiaeorientlis*) rely on clicks for navigation while traveling and foraging, and they have been shown to produce click trains every 5.1 s on average [6], with a click train rate varying from 0 to 290 click trains per 10 min [39]. Yangtze finless porpoise clicks are similar to those of other members of the *Phocoenidae* family or of *Cephalorhynchus* spp. within delphinids: they are typical high-frequency narrow-band ultrasonic pulses with peak frequencies ranging between 87 and 145 kHz [54]. These freshwater animals suffer from greater threats than other species, including being trapped in shallow areas, intense anthropogenic disturbances increasing risks of entanglement with underwater debris or fishing nets, or collision with boats. The environment they live in (i.e., turbid water) does not enable them to efficiently use their vision, making echolocation even more crucial for these animals’ survival. Because of the difficulty of observing Yangtze finless porpoises in their natural habitat, population density is often assessed using passive acoustic monitoring that relies on counting the number of click trains [55,56,57]. Because of anthropogenic threats, Yangtze finless porpoises are now critically endangered [58,59], and in order to add to the in situ conservation efforts, porpoises are being held in captivity or semi-natural environments [59]. The acoustical adaptability of these animals to different environments has rarely been studied. The only work that investigated such abilities involved three groups of Yangtze finless porpoises living under different conditions (i.e., captivity, reserve, wild) and revealed differences in their click production, suggesting that they would adapt their echolocation signals depending on their living conditions [60]. This result has shown that animals modulate their acoustical activity depending on the environment they live in, but within each environment, the context-induced variations in porpoise click production have never been studied. In the present study, we recorded the acoustic activity of a group of five captive Yangtze finless porpoises and analyzed their click production variations depending on the context. The aim of this study was to describe click rate patterns in this group to investigate whether Yangtze finless porpoises modulate their click production depending on the context. The usefulness of this easy-to-assess parameter as a tool to monitor captive porpoise welfare was then considered.

## 2. Materials and Methods

### 2.1. Study Animals and Captive Management

**Figure 1:**
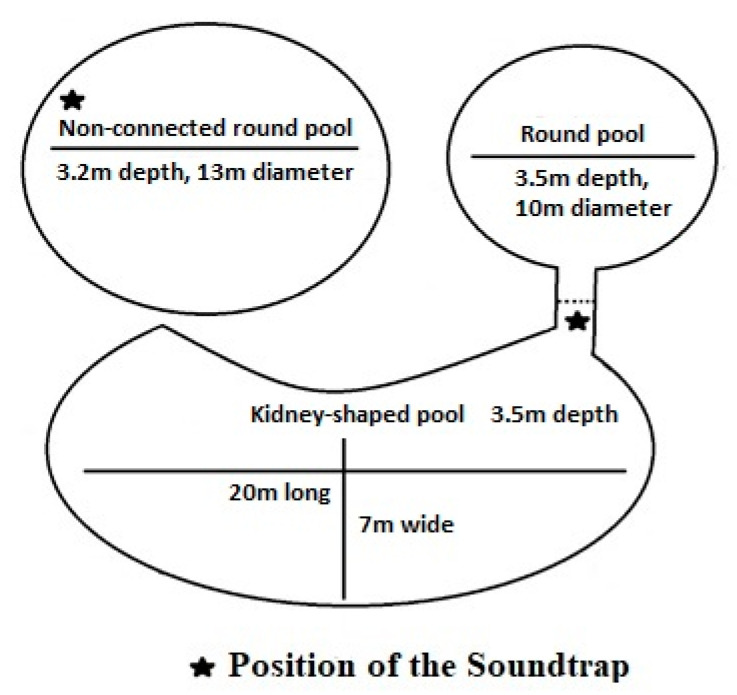
Design of the housing pools and position of the Soundtrap in both pools.

Data were collected from October 2017 to November 2018. The study subjects were five Yangtze finless porpoises housed in Baiji Dolphinarium, Institute of Hydrobiology, Chinese Academy of Sciences (Table 1). Porpoises were usually housed in a two-pool complex including a kidney-shaped pool, a connected round pool, and a non-connected round pool (Figure 1). The social grouping changed several times during data collection. When social separation occurred, a gate between the two connected pools was closed, still allowing visual and acoustical contact. The three females were pregnant during the data collection, and gave birth during summer 2018. A third pool was used to house the female F7 and the male Taotao from February 2017, until F7 gave birth (2 June 2017). When the female F7 gave birth, she remained alone in this pool with her calf and Taotao was moved back into the two-pool complex. Two calves were present from their birth to their death (two weeks after their birth), and the third one (F7′s calf) was present from its birth until the end of data collection.

Porpoises were fed between 3 and 3.5 kg of thawed Basilewsky fish (*Siniperca chuatsi*) and/or live fish per day during four to six training/feeding sessions. The total amount of fish provided per day to each animal varied with age, sex, season, and reproductive status, and was designed to maintain a healthy body weight and optimum condition. Occasional visitors were allowed to watch animals both from the surface and from underwater windows. Visitors were groups of between two and thirty persons (4.57 ± 5.99 persons on average), and visits were planned randomly (usually not more than one visit per week). Enrichment was provided (toys, live fish, or free interaction with caretakers). Pools were cleaned by divers and/or caretakers scrubbing the upper part of the pools’ walls approximately once per month.

### 2.2. Data Collection

A Soundtrap 300 HF (Ocean Instruments Ltd., Whangateau, New Zealand) was used to record underwater sound. The sampling rate was set at 576 kHz, the number of analog-to-digital bits was set at 16 bit, and high-pass filtering was turned off. Animals had been in contact with the Soundtrap from September 2017, and were therefore habituated to it and rarely explored it or interacted with it. The Soundtrap was tied to a rope and placed in the corridor between the two connected pools or on a side of the pool in the non-connected pool, at approximately 2 m depth. Data were collected for a minimum of two days a week, and the Soundtrap was usually placed in the pool in the early morning and removed in the late afternoon in order not to create a change in the porpoises’ environment right before recording. When a group was housed in the non-connected pool, and one in the two-pool complex, the Soundtrap was randomly placed in one of the two pools for the entire day, or was placed in one pool in the morning and in the other at noon. Each recording day, a minimum of three 10–20 min samples were extracted from the Soundtrap: usually one in the morning, one at noon, and one in the afternoon. The precise sampling time depended on the day’s schedule, which was never exactly the same.

For each of the 10–20 min samples, the associated conditions were noted, including: season, time of day, time relative to training/feeding sessions, social grouping, presence of enrichment, unusual events, and presence of visitors (Table 2).

### 2.3. Analysis

#### 2.3.1. Echolocation Signal Analysis

Data were split into one-minute recordings. MATLAB R2017a was used to analyze each one-minute sample. Here, following previous studies, we defined porpoise echolocation signals as clicks [3] ranging between 87 and 128 kHz [54]. Because of the number of individuals present and the potential reflection of the signals they produced by the water surface and pool walls, records often contained a large number of clicks. These clicks were usually produced in trains defined as a minimum of five consecutive clicks [57,61], with a maximum of 200 ms between clicks (inter-click interval, [6,62]). The resulting large number of click trains recorded were often overlapping or very close to each other. Using MATLAB to analyze click trains was therefore a difficult task; it was determined that only clicks themselves but not click trains would be analyzed to keep the analysis as simple as possible so that captive facilities could easily reproduce it and use it to monitor their animals. The MATLAB algorithm was based on a support vector machine (SVM) model, one of the binary classification machine-learning methods, to distinguish clicks. A first-order finite impulse response (FIR) high-pass filter was used to filter the signal, with a Kaiser window function (beta = 0.5) and an 80 kHz cut-off frequency. After this first step, a short-time Fourier transform (window = 512, noverlap = 256, nfft = 512) was applied to obtain the time-frequency distribution of the signal. Then a dataset consisting of 2000 spectra of representative clicks and background noise was created. Random noise was also added within 3dB to ensure the generalization performance of the dataset. The SVM model was performed after data enhancement, and an accuracy rate of 98.7% through 5-fold cross-validation was eventually achieved. The goal here was not to obtain the exact number of clicks produced by the animals or their characteristics, but to analyze variations in their production. Even if the number of clicks obtained through the analysis was overestimated because of the reflections, these reflections were assumed to always be the same (same pool); therefore, variations in the number of clicks recorded reflected the actual variation in Yangtze finless porpoise click production. To ensure the accuracy of the MATLAB analysis, a visual/manual analysis was additionally conducted on 10% of the data. Number of clicks per minute will hereafter be referred to as “click density”.

#### 2.3.2. Statistical Analysis

All statistical analyses were performed using R 4.0.3. The effect of the number of individuals present in the pool, and of the environmental and social variables on the click density was analyzed by fitting a generalized linear mixed-effects model (GLMM) for Poisson distributed data using the “glmer” function from the “lme4” package [63]. A bound optimization by quadratic approximation (“bobyqa”) optimizer that performs derivative-free bound-constrained optimization using an iteratively constructed quadratic approximation for the objective function was added. For this model, the click density was the response variable, the number of individuals present in the pool was included as an offset to standardize the density, and the date and observation session ID were included as random factors. The predictors included number of individuals present, time of day, time relative to training, social grouping, presence of enrichment, unusual events, and presence of visitors. A model diagnosis was conducted, including a test for overdispersion and a test for collinearity. Collinearity was tested by looking at variables’ variance inflation factor (VIF). Because the enrichment variable caused collinearity issues (VIF > 3), this variable was split into four variables (presence of toys, presence of humans, presence of toys and humans together, presence of new objects). The live fish enrichment was excluded from the analysis because of a too small sample size. An additional case level random factor was added to correct over-dispersion. The model with the lowest Akaike information criterion (AIC) was selected [64]. A Wald chi-squared test was conducted to extract *p*-values from the model. Pairwise tests were conducted by running the selected model with an appropriate sub-setting (i.e., same model, with only two levels of the targeted predictor included) and a sequential Bonferroni correction.

## 3. Results

**Figure 2:**
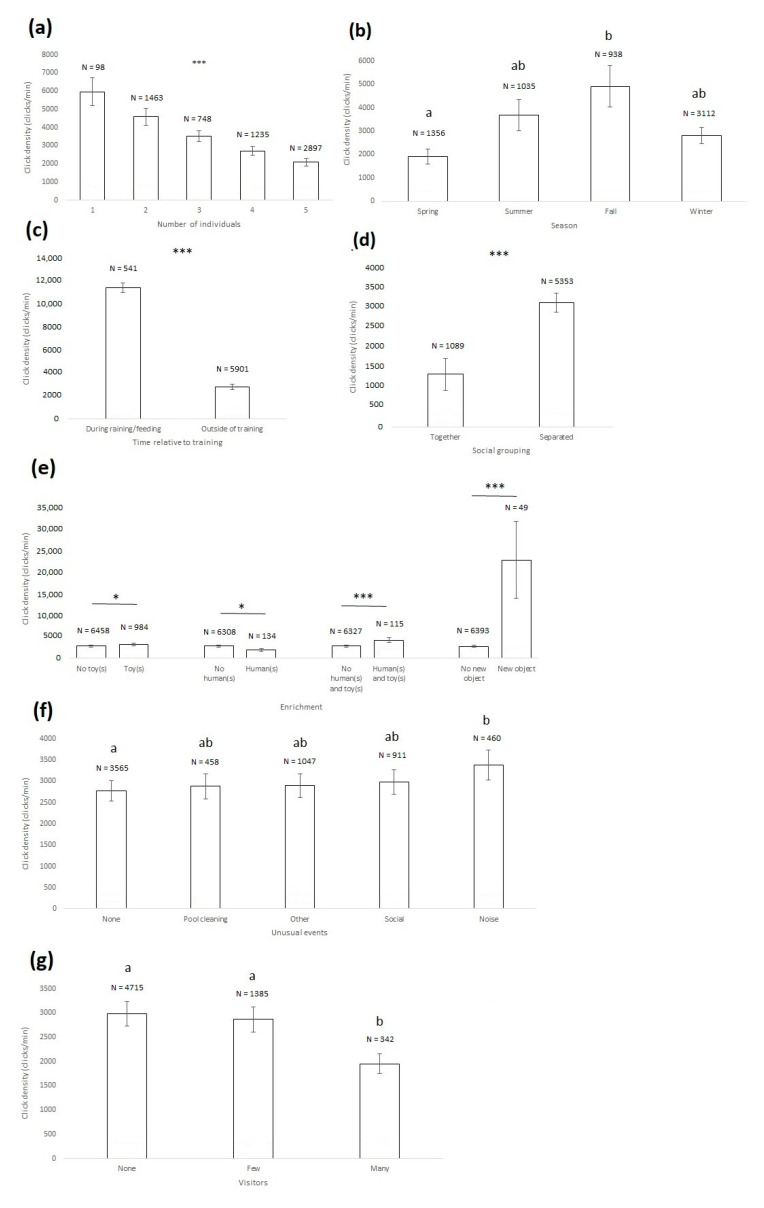
Click density average and 95% confidence intervals depending on the number of porpoises present in the pool (**a**) the season (**b**) the social grouping (**c**), the presence of enrichment (**d**) the time relative to training (**e**) the occurrence of unusual events (**f**) and the presence of visitors (**g**) *: *p* < 0.05, ***: *p* < 0.001; when pairwise tests were conducted within a context variable, click densities that share the same letter (a or b) do not differ significantly, whereas click densities that have no letter in common differ significantly (Wald chi-squared test with a sequential Bonferroni correction). N: number of one-minute samples. The presented data are not raw, but have been extracted from the generalized linear mixed-effects model.

A total of 6442 min (approximately 107 h) of recording was analyzed. The click density ranged between 0 and 9576 clicks per minute (3864.33 ± 2189.60 clicks on average). The click density (i.e., number of clicks per minute) was negatively linked to the number of porpoises present in the pool (Figure 2a, Table 3). The more porpoises in the pool, the less clicks were produced. The click density was significantly affected by the season: it was significantly higher in fall than in spring (Figure 2b, Table 3). The click density did not significantly vary depending on the time of day, but it was significantly higher during training/feeding sessions than outside of such sessions (Figure 2c, Table 3). Clicks were significantly more frequent when porpoises were separated by a gate than when not (Figure 2d, Table 3). Clicks were significantly more frequent with the presence of toys, humans and toys together, and new objects, but less frequent when humans were present alone than when not present (Figure 2e, Table 3). Clicks were significantly more frequent during noisy events rather than when no unusual event occurred (Figure 2f, Table 3). The presence of visitors significantly impacted the click density, with less clicks produced when many visitors were present rather than when few or none were (Figure 2g, Table 3).

## 4. Discussion

First, the standardized number of clicks decreased with increasing number of animals present in the pool. Clicks are thought to serve both as an echolocation and a communication function in odontocetes such as Yangtze finless porpoises that do not whistle [9,65,66,67]. With more animals present, each animal’s clicks are added to the others’, and we expected a higher occurrence of clicks. A low number of animals in a pool always followed a separation and transport process during which porpoises were lifted out of the water and moved to a new pool. During this process, they were separated from some of their congeners. They might have needed to (1) explore this new environment, and (2) locate the congeners that stayed in the initial pool, resulting in more clicks. This hypothesis requires further investigation.

Unlike previous findings suggesting that clicks, which are used mainly to navigate, feed, and communicate, were unlikely to exhibit any seasonal cycle (e.g., [68]), a seasonal pattern was found in the Yangtze finless porpoises’ click production. Yangtze finless porpoises are seasonal breeders, and their socio-sexual activities increase in summer and fall [69]; such periods may require more communication as well as more echolocation to locate potential mates. Conversely, the click density did not exhibit any diurnal pattern. Some studies have shown that wild Yangtze finless porpoises produced more clicks at night than during the day, suggesting that these animals were feeding at night [70], while other studies found no day/night differences [39]. Data were not collected at night in this study, and only differences between morning, noon, and afternoon were analyzed. Such analysis could bring additional information about the diurnal rhythm of Yangtze finless porpoises’ acoustic production.

Social changes are likely to impact odontocete sound production [43]. Captive bottlenose dolphins have been shown to whistle 7.8 times more in isolation than when they were not separated from their congeners [71], and social separation has been reported to affect some whistle types but not others in this species [43]. Yangtze finless porpoises produced more clicks when they were separated in subgroups. Being physically separated into two pools with a gate and yet remaining in acoustic contact, Yangtze finless porpoises might have used clicks in order to communicate and maintain cohesion between pools, or to try to locate other individuals through the gate.

Yangtze finless porpoises produced more clicks during training/feeding sessions than outside of such sessions. This pattern has already been reported in other captive odontocetes with an increase in all types of underwater sounds during training/feeding/public presentations (bottlenose dolphins [42,72,73,74,75]; belugas, *Delphinapterus leucas* [76]; Pacific white-sided dolphins, *Lagenorhynchus obliquidens* [77]). Such routine events might have caused an increased level of excitement or anticipation [72,73]. In addition, studies have shown that odontocete click rate increases when foraging because they produce buzzes that are click trains with short inter-click intervals [8,78,79,80]. Yangtze finless porpoises have been shown to emit such buzzes when feeding, especially when an individual catches its prey [69]. During training/feeding sessions, the presence of prey (both dead and live), and the animals’ eating behavior may have elicited a production of buzzes which resulted in an increased click density. Porpoises could also have simply increased their echolocation activity and produced more clicks when prey were present. The fact that the Yangtze finless porpoises’ click density was the highest when live fish were present (compared with other types of enrichment) is congruent with this hypothesis.

Clicks were also more frequent when toys, humans and toys, or new objects were present together rather than when not. The presence of humans has been shown to be linked to an increased sound production in captive odontocetes [43,74,77], which could explain why the presence of humans and toys together resulted in a higher click density than when toys were provided alone. However, the presence of humans alone resulted in a lower click density. When humans were present together with toys, these humans were always familiar and they always tried to interact with the porpoises (e.g., showing them familiar or new toys, throwing them in the pool), and porpoises often participated in the interaction (e.g., approaching, observing, touching, bringing objects back). In addition, these familiar humans were usually caretakers, and porpoises might have associated their presence with events such as training sessions and feeding; an increase in clicks production may therefore have been linked to an anticipation of such events when caretakers were interacting with them. When caretakers were present without toys, porpoises were often coming to observe them and leaving as soon as they realized no fish or toys were provided. This reaction could be an explanation for the lower click density associated with human enrichment, but more work is needed to validate this hypothesis. The presence of many visitors was also linked to a lower production of clicks. Visitors were not allowed to approach the water surface, and porpoises interacted much less with them. Such infrequent and often noisy stimulus might have been stressful for the animals, and producing less clicks may be a response to this stressful stimulus. Regarding the presence of new objects in the pool, the higher density of clicks may be linked to an increased exploration behavior.

Yangtze finless porpoises’ click production was higher during noisy events (e.g., construction work, unusual noisy meetings) than when no unusual event occurred. When discussing the effects of boats on wild odontocetes, it was suggested that a boat’s physical presence, its noise, and its behavior together might impact the animals’ perception of risk to determine their response [81,82]. Because it is often hypothesized that odontocetes are sensitive to noise [83,84], noisy events have the potential to affect their behavior, including vocalizations. The porpoises in this study may have used clicks to search for and investigate the source of the noise. Social events and pool cleaning did not significantly influence Yangtze finless porpoise click production. The few studies that investigated the impact of unusual events that occur in captive facilities on the acoustic activity of odontocetes reported a decreased acoustic production in beluga whales after transportation to a new facility and after the introduction of four harbor seals (*Phoca vitulina*) into their pool [52], and a decrease in acoustic productions during pool cleaning for bottlenose dolphins [43]. Here, social events were group separations and reunions, and it seems that Yangtze finless porpoises did not change their click production much following these events. Pool cleaning, which elicits changes in behavior in this group of Yangtze finless porpoises [85,86,87], did not significantly influence their click production either. Noise and pool cleaning can represent stressful stimuli, noise being an acoustic one, while pool cleaning involves the presence of moving objects/humans in the water. The fact that Yangtze finless porpoises modified their click production during noisy events but not during pool cleaning suggests that different types of potentially stressful stimuli elicit different responses, and that behavior should be monitored together with acoustical activity to get a full picture of the animals’ reaction.

Although studies investigating the link between vocalizations and welfare in odontocetes are scarce, previous studies have suggested that variations in odontocete signals might be related to emotional states and specific events [88,89,90]. Most studies focused on the link between behavior/context (e.g., excitement, bow riding, feeding, socializing) and wild animal whistle production (Hawaiian spinner dolphins, *Stenella longirostris* [27]; common dolphins, *Delphinus delphis* [91]; pilot whales, *Globicephala sp.* [92]; bottlenose dolphins [93,94]). For porpoises that are thought to only produce one type of vocalization (high-frequency clicks), it has been suggested that differences in click train characteristics (e.g., inter-click interval) are dependent on behavioral context [9,68,95]. Here, because of the reflection phenomenon, we could not analyze click trains and their parameters.

However, despite these reflections, the variations we observed in click density seemed to be reliable and to indicate which variables influenced Yangtze finless porpoises’ click production and in what ways. The variations observed depending on the number of animals present and on the presence of prey allow us to validate the patterns we found for the other variables. Among these other variables, social separation, presence of enrichment, presence of visitors, and potentially stressful events probably resulted in changes in the animals’ emotional state, and the changes in click production we observed might be indirectly reflecting these emotional state changes. The patterns we found might help the care team to understand their animals’ acoustical activity and to adapt their management to this activity. An abrupt change in click density after a management decision such as group separation or the occurrence of any kind of unusual event should be alerting and trigger a closer monitoring of the animals to ensure they are adapting adequately to the situation for instance. Also, starting a training session when porpoises’ click production is relatively high might not be very efficient as the animals might not be fully attentive, but this hypothesis needs further work to be validated. It seems that the relationship between click production and emotional state can be ambiguous, and hard to interpret on its own. For instance, click density is particularly high when porpoises are feeding, which can be seen as a positive situation, but it is also very high when animals are socially separated, which is probably experienced as a negative situation by these porpoises [85,86,87]. Noting the context when collecting data is crucial to avoid misinterpretations. Additionally, because some variables may influence acoustic production while others may influence behavior, and because behavioral data may help the interpretation of acoustical data, monitoring captive animals’ click production should be combined with behavioral observations.

In addition to these potential applications in captive settings, our study also provides information that can be used when monitoring wild populations. Such information on the contexts in which captive Yangtze finless porpoises increase or reduce their click production could aid in understanding wild animal responses to anthropogenic disturbances. A low click density could reflect not only the absence or low number of porpoises in a zone, but also their low click production in response to a noisy environment. In addition, the important click density difference when animals are eating/hunting versus when they are not should be considered when using clicks as an indicator of Yangtze finless porpoise density. Foraging animals and resting/traveling/socializing animals do not produce the same number of clicks, and the number of Yangtze finless porpoises present may be over- or underestimated. However, the click production of wild and captive individuals may differ, and studying the differences in click rates between wild and captive porpoises as well as the variation of click rates depending on potential wild disturbances is needed to determine if results obtained in captivity can be applied in the wild. Considering the different environments in which wild and captive porpoises live, their click rates may not vary depending on similar factors or following similar patterns for instance. We therefore suggest that more research should be conducted to investigate the acoustical response of Yangtze finless porpoises to various stimuli, in both wild and captive settings, and that other kinds of signals should be analyzed (e.g., burst-pulsed signals).

## Figures and Tables

**Table 1:** Catalog of studied individuals’ features.

Name	Sex	Age (year)	Length (m/h)
**Duoduo**	M	8	157
F7 *	F	8	145
F9 *	F	8	145
**Taotao**	M	14	156
Yangyang *	F	11	147

* pregnant females that gave birth during the data collection.

**Table 2:** Context variables.

Season	Time of Day	Time Relative to Training *	Social Grouping		Enrichment	Unusual Events	Visitors
Winter (December–February)	Morning (8 am–11 pm)	Outside of training	Together		None	None	None
Spring (March–May)	Noon (11 pm–2 pm)	During training	Separated		Toys ^a^	Pool cleaning ^d^	Few ^h^
Summer (June–August)	Afternoon (2 pm–5 pm)				Humans ^b^	Noise ^e^	Many ^i^
Fall (September–November)					Humans and toys	Social event ^f^	
					Live fish	Other event ^g^	
					New object ^c^		

* Training: training or feeding session. ^a^ Balls. ^b^ Caretakers or other humans interacting with porpoises from the surface or from underwater windows. ^c^ Stretcher, material used for new experiments, new toys. ^d^ Diver and/or caretaker scrubbing the surface using long-handle brushes. ^e^ Construction work or people having meetings next to the pool to prepare events such as transport of animals. ^f^ Right after separation or reunion of groups (or separation attempts for BDs). ^g^ Shoal of small fish in the pool, unusual water level. ^h^ <5 persons next to the pool: employees or visitors. ^i^ >5 persons next to the pool: visitors.

**Table 3:** Model characteristics (a) and statistical outputs (b) from the generalized linear mixed-effects model.

(a)	Model Components				Model Characteristics					Scaled Residuals				
	Response Variable	Offset	Random	Predictors	AIC	BIC	logLik	Deviance	df.resid	Min	1Q	Median	3Q	Max
			factors											
Selected model	Click density	Number of individuals	Date,	Number of individuals,	107,583.40	107,717.10	−53,771.7	107,543.40	5878	−4.1621	−0.0065	0.0019	0.0093	1.0357
			Observation session ID	Season,										
				Time relative to training,										
				Social grouping,										
				Presence of toys,										
				Presence of humans,										
				Presence of humans and toys,										
				Presence of new objects,										
				Unusual events,										
				Presence of visitors										
**(b)**	**Number of individuals**	**Season**	**Social grouping**	**Presence of toys**	**Presence of humans**		**Presence of humans and toys**		**Presence of new objects**		**Time relative to training**		**Unusual events**	**Presence of public**
Selected model	χ^2^ = 48.687, df = 1, *p* < 0.0001	χ^2^ = 17.076, df = 3, *p* = 0.0007	χ^2^ = 18.709, df = 1, *p* < 0.0001	χ^2^ = 5.256, df = 1, *p* = 0.0218	χ^2^ = 6.580, df = 1, *p* = 0.0103		χ^2^ = 13.205, df = 1, *p* = 0.0003		χ^2^ = 30.067, df = 1, *p* < 0.0001		χ^2^ = 617.822, df = 1, *p* < 0.0001		χ^2^ = 8.915, df = 4, *p* = 0.0634	χ^2^ = 40.891, df = 2, *p* < 0.0001

## Data Availability

The data presented in this study are available on request from the corresponding author.
